# β-Ionone Treatment Enhances the Antioxidant Capacity in Postharvest Broccoli (*Brassica oleracea* L. var. *Italica*) by Maintaining the Levels of Bioactive Substances

**DOI:** 10.3390/foods14050762

**Published:** 2025-02-24

**Authors:** Feng Zhang, Mengze Cao, Letong Shen, Liyu Shi, Wei Chen, Zhenfeng Yang

**Affiliations:** 1 College of Food Science and Technology, Shanghai Ocean University, Shanghai 201306, China; 18625566539@163.com; 2 Zhejiang Key Laboratory of Intelligent Food Logistic and Processing, College of Biological and Environmental Sciences, Zhejiang Wanli University, Ningbo 315100, China; slt409186506@163.com (L.S.); shiliyu@zwu.edu.cn (L.S.); 3 Seymour College, Glen Osmond, SA 5064, Australia; mcao.65126@seymour.sa.edu.au; 4 Zhejiang Key Laboratory of Intelligent Food Logistic and Processing, Hwamei College of Life and Health Sciences, Zhejiang Wanli University, Ningbo 315100, China; vivancanyee@zwu.edu.cn

**Keywords:** β-ionone, broccoli, bioactive substances, antioxidant capacity

## Abstract

Broccoli is prone to nutrient loss during postharvest storage due to its high respiratory metabolism. In this study, we investigated the effects of 0.1 mm β-ionone on bioactive substances and antioxidant capacity during postharvest storage of broccoli. We found that the decline in the scavenging rates of 1,1-diphenyl-2-picrylhydrazyl and 2,2′-azinobis-(3-ethylbenzthiazoline-6-sulphonate) radicals was delayed in the treated florets. This delay is attributed to β-ionone treatment, which upregulated the expression of biosynthetic genes related to glucosinolates and riboflavin in broccoli, thereby slowing the loss of these nutrients. Additionally, β-ionone treatment increased the transcript levels of anabolic genes while reducing the expression of genes encoding enzymes involved in the catabolism of ascorbic acid (AsA) and glutathione (GSH), resulting in higher levels of AsA and GSH in treated broccoli compared to the control. Overall, β-ionone treatment enhanced antioxidant capacity by delaying the loss of bioactive substances in postharvest broccoli. These findings provide the first evidence that exogenous β-ionone helps preserve antioxidant capacity in postharvest horticultural products.

## 1. Introduction

Broccoli (*Brassica oleracea* L. var. *italica*) is favored by consumers for its crisp texture and refreshing flavor. It has garnered increasing attention due to its rich content of bioactive substances such as proteins, glucosinolates, riboflavin, AsA, and GSH [1]. However, during postharvest storage, broccoli’s vigorous respiratory metabolism can easily lead to the loss of these nutrients [2].

Glucosinolates are important secondary metabolites in broccoli, acting as bioactive components, with significant roles in antioxidation, neuroprotection, cancer prevention, and endocrine regulation [3]. Research has revealed that glucoraphanin (GRA), glucobrassicin (GBS), and neoglucobrassicin (NGBS) make up 92% of the total glucosinolate content in broccoli, which decreases sharply during storage [4]. Riboflavin is a crucial water-soluble nutrient found in living organisms, mainly present as flavin mononucleotide (FMN) and flavin adenine dinucleotide (FAD). It is involved in many metabolic redox reactions and plays an important role in regulating plant growth and development [5]. AsA is present in most plants and tissues, playing a crucial role in energy metabolism, growth, development, and redox reactions [6]. GSH, a tripeptide containing γ-amide and sulfhydryl groups, forms a non-enzymatic antioxidant system together with AsA [7]. During the postharvest storage of broccoli, the production of reactive oxygen species (ROS), including superoxide anion (O_2_^−^), hydrogen peroxide (H_2_O_2_), hydroxyl radical (OH^−^), ozone (O_3_), and singlet oxygen (^1^O_2_), is closely linked to oxidative stress responses. Proteins, lipids, and other nutrients in broccoli can be damaged by the production of ROS [8]. Antioxidants such as glucosinolates [9], riboflavin [10], AsA, and GSH [11] can quickly reduce ROS levels, protecting tissue integrity. Specifically, the AsA-GSH cycle can reduce H_2_O_2_ to H_2_O through the oxidation of AsA under enzymatic action. The oxidation product reacts with GSH to regenerate AsA, producing GSSG, which can be catalytically reduced back to GSH. This cycle of AsA and GSH continuously removes H_2_O_2_, reducing ROS levels [12]. The loss of these bioactive substances severely affects the nutritional and market value of broccoli. Therefore, exploring protective techniques is of great practical significance.

β-ionone is a cleavage product of carotenoids, widely present in plants and known as a natural compound. As a cyclic isoprenoid, it is extensively used in industry for the synthesis of compounds such as retinoic acid, retinol, β-carotene, and vitamin A [13]. It has been approved by the United States, the European Commission, and various other countries and organizations for use as a food additive and fragrance in the food and cosmetics industries [14]. Additionally, due to its excellent anticancer, anti-inflammatory, and antioxidant properties, β-ionone is widely used in medical research. For instance, β-ionone has been demonstrated to activate the prostate-specific G-protein-coupled receptor (PSGR), which in turn inhibited the proliferation of prostate cancer cells [15]. In the case of liver cancer, β-ionone is a promising chemopreventive agent, which can reduce the potential targets for the development of hepatocellular carcinoma cells [16]. In recent years, exogenous β-ionone has also been used to enhance resistance against gray mold in Arabidopsis and tomatoes by regulating the levels of abscisic acid and the expression of antimicrobial-related genes [17]. However, no information on the application of β-ionone for postharvest preservation of fruits and vegetables is available. Therefore, the effect of β-ionone treatment on the contents of bioactive substances and antioxidant capacity in postharvest broccoli was investigated to provide new technical support for its postharvest preservation.

## 2. Materials and Methods

### 2.1. Plant Materials and Treatment

Fresh broccoli was harvested from a commercial plantation in Cixi City, China, approximately 110 days after sowing. After the harvest, the broccoli was transported to the laboratory within two hours. Broccoli, with a weight of about 400 g, a uniform dark green color, consistent bud size, and free from insect or mechanical damage, was selected as experimental material and was divided into two groups, each containing 40 broccoli. Our preliminary experiments investigated the effects of various β-ionone concentrations (0.05 mm, 0.1 mm, 0.5 mm, and 1 mm) on the antioxidant activity of broccoli. The results indicated that 0.1 mm β-ionone was the most effective in preserving the antioxidant properties of broccoli. Consequently, this concentration was selected for subsequent treatments. Under the same experimental conditions, the two groups of broccoli florets were, respectively, immersed in a 0.1 mm β-ionone solution and distilled water (both containing 1% anhydrous ethanol) for 10 min, with the distilled water treatment group serving as the control. After the natural drying of surface moisture, the broccoli florets were stored in a controlled environment chamber with a temperature of 15 °C and a relative humidity of 90% for a total of four days. During the storage period, sampling was conducted daily, with 8 broccoli florets being sampled from each group each time, and their buds collected for three biological replicates. Additionally, daily photographs were taken throughout the storage period, and the images of broccoli florets are provided in Supplementary Figure S1. The entire experiment was repeated three times. The broccoli florets were then rapidly frozen in liquid nitrogen and stored at −80 °C for subsequent analysis.

### 2.2. Determination of Antioxidant Capacity

The antioxidant capacity of broccoli was assessed following Suo et al. [18] with minor adjustments. 1.0 g samples were ground in liquid nitrogen and subsequently mixed with 10 mL of an 80% methanol solution. Ultrasonic-assisted extraction was conducted at 25 °C, using a frequency of 35 kHz and a power density of 40 W L^−1^ for 25 min. Subsequently, the sample was subjected to centrifugation at 5000× *g* for a period of 5 min at 4 °C to obtain the supernatant. For the 1,1-Diphenyl-2-picrylhydrazyl (DPPH) assay, 1 mL of the supernatant was mixed with 4 mL of 0.2 mm DPPH solution. For the 2,2′-Azinobis-(3-ethylbenzthiazoline-6-sulphonate) (ABTS) assay, 1 mL of the supernatant was combined with 8 mL of 0.1 mm ABTS solution. Both mixtures were incubated in the dark at 25 °C for 40 min. Absorbance was measured at 517 nm for DPPH and 734 nm for ABTS. The free radical scavenging rate was then calculated according to the methodology [18]. Three biological replicates were performed.

### 2.3. Determination of Glucosinolate Content

Based on the report by Yang et al. [4], the main glucosinolate components in broccoli are GRA, GBS, and NGBS. Therefore, the total glucosinolate content in broccoli was evaluated through the measurement of the aforementioned three components.

Here, 0.5 g samples were combined with 4 mL of 70% (*v*/*v*) methanol solution and 0.2 mL of an internal standard solution (5 mm sinigrin) in a 15 mL centrifuge tube. Following a period of vertexing for a duration of one minute, the mixture was then incubated in a water bath maintained at a temperature of 70 °C for a period of 30 min. Ultrasonic-assisted extraction was conducted at 4 °C, using a frequency of 35 kHz and a power density of 40 W L^−1^ for 25 min. The sample was subjected to centrifugation at 3000× *g* for 20 min at 4 °C to prepare the supernatant. The supernatant was then collected and evaporated to near dryness with a nitrogen blower at 35 °C. Subsequently, the residue was re-dissolved in 1 mL of ultrapure water. Subsequently, the solution was filtered through a 0.22-µm microporous membrane for subsequent analysis.

The samples were subjected to analysis using high-performance liquid chromatography-tandem mass spectrometry. The content was determined using the internal standard method based on relative response factors [19]. A Waters ACQUITY UPLC H-CLASS system (Waters, Milford, MA, USA), comprising an ACQUITY UPLC HSS T3 column (2.1 × 100 mm, 1.8 µm), was utilized for chromatographic separation purposes. The mobile phases comprised 0.2% (*v*/*v*) formic acid in water (A) and acetonitrile (B). The elution program was as follows: 0–0.5 min, 95% A; 3 min, 60% A; 3.5–4.0 min, 5% A; 4.5–6.0 min, 95% A. A flow rate of 0.2 mL min^−1^ was applied, with an injection volume of 2 µL. The temperature of the column was maintained at 350 °C. The liquid chromatography-mass spectrometry system (SYNAPT G2-Si, Waters) was equipped with an electrospray ionization source (ESI-) operating in negative ion scan mode and multiple reaction monitoring (MRM) mode for the quantification of target compounds. The temperature of the electrospray source was set at 120 °C, the temperature of the desolvation was set at 350 °C, and the voltage for ionization was set at 3000 V. Three biological replicates were conducted.

### 2.4. Determination of Riboflavin Content

Riboflavin extraction was conducted using a modified version of the approach described by Chen et al. [20]. A sample weighing 0.2 g was mixed with 1 mL of an 80% methanol extraction solution (containing 1% acetic acid) and homogenized. Ultrasonic-assisted extraction was conducted at 4 °C, using a frequency of 35 kHz and a power density of 40 W L^−1^ for 20 min. Subsequently, the samples were subjected to centrifugation at 5000× *g* for a period of 10 min to obtain the supernatant. After the initial collection of the supernatant, an additional two rounds of extraction were performed. Finally, the resulting supernatant was filtered through a 0.45 µm organic phase microporous membrane for subsequent analysis.

The samples were subjected to analysis using high-performance liquid chromatography-tandem mass spectrometry. Riboflavin was employed as the standard, with quantification conducted via the external standard method [21]. A Waters ACQUITY UPLC H-CLASS system (Waters), comprising an ACQUITY UPLC HSS T3 column (2.1 × 100 mm, 1.8 µm), was utilized for chromatographic separation purposes. The liquid chromatography parameters were as follows: mobile phase A was ultrapure water (containing 0.1% acetic acid), and mobile phase B was acetonitrile (containing 0.1% acetic acid); flow rate was 0.3 mL min^−1^; column temperature was 40 °C; injection volume was 2 µL. The elution gradient comprised the following steps: 0 min A/B (90:10 *v*/*v*), 1.0 min A/B (90:10 *v*/*v*), 5.0 min A/B (10:90 *v*/*v*), 7.0 min A/B (10:90 *v*/*v*), 7.1 min A/B (90:10 *v*/*v*), and 9.0 min A/B (90:10 *v*/*v*).

The analysis was conducted on a liquid chromatography-mass spectrometry system (SYNAPT G2-Si, Waters) utilizing the following electrospray ionization (ESI) conditions: an ion spray voltage of 3000 V, a temperature of 350 °C, and an ion transfer tube temperature of 320 °C. The selected scan mode was ion monitoring (SIM), and the scan type was positive ion mode. Three biological repeats of the assay were performed.

### 2.5. Determination of AsA Content

A standard AsA solution (1 mg mL^−1^) was prepared with 5% (*w*/*v*) trichloroacetic acid as the solvent. Subsequently, 0.5 mL of a phosphate solution with an ethanol concentration of 0.4%, 1 mL of a phenanthroline solution with an ethanol concentration of 0.5%, and 0.5 mL of an FeCl_3_ solution with an ethanol concentration of 0.03% were added in sequence via gradient dilution. Mix and place the mixture in a water bath at 30 °C for 65 min. Absorbance was measured at 534 nm to create a standard curve. For sample preparation, 0.1 g of the sample was pulverized in a nitrogen bath and combined with 5 mL of 5% trichloroacetic acid. The homogenate was subjected to centrifugation at 5000× *g* for 15 min at 4 °C, thereby obtaining the supernatant. One milliliter of the supernatant was used to measure absorbance according to the AsA standard method, and the concentration was determined from the standard curve [22]. Three biological replicates were conducted.

### 2.6. Determination of GSH and GSSG Content

0.1 g powder samples ground in liquid nitrogen were taken for the measurement of GSH and GSSG levels using a glutathione assay kit (Solarbio, Beijing, China). Three biological replicates were performed.

### 2.7. Analysis of Gene Expression in Broccoli

The extraction and reverse transcription of total RNA were carried out in accordance with the methodology described by Yan et al. [23]. The RT-qPCR reaction was performed using SYBR Green I Premix (Vazyme, Nanjing, China). Specific primers were designed using Beacon Designer 7 software (listed in Supplementary Table S1), with *BoActin* as the internal reference gene. The real-time quantitative PCR instrument used was the Step One PlusTM (Thermo Fisher Scientific, Waltham, MA, USA). Four biological replicates were set up, and relative gene expression levels were determined through the use of the 2^−ΔCT^ method.

### 2.8. Data Analysis and Statistics

Statistical analysis was performed using GraphPad Prism 8 software. Experimental results are presented as mean ± standard deviation. A two-way analysis of variance (ANOVA) was conducted to assess the effects of treatment and storage time, followed by Tukey’s test for multiple comparisons. Differences were considered statistically significant at *p* < 0.05. The normality of the residuals and homogeneity of variances were verified using the Shapiro–Wilk test and Bartlett’s test, respectively, with the results presented in Supplementary Tables S2 and S3.

## 3. Results

### 3.1. Effects of Β-Ionone Treatment on DPPH and ABTS Radical Scavenging Rates in Postharvest Broccoli

**Figure 1:**
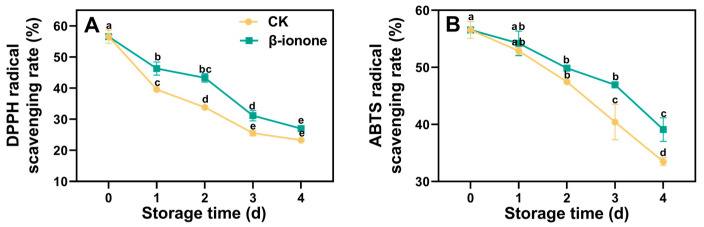
The DPPH (**A**) and ABTS (**B**) radical scavenging rates of broccoli treated with β-ionone and the control during storage at 15 °C. Mean values (n = 3) and standard deviation (vertical bars) are represented. Data points carrying different letters indicate statistically significant differences (*p* < 0.05).

As shown in Figure 1, the rate at which the DPPH and ABTS radicals in broccoli were scavenged decreased progressively over time during storage. The treatment with β-ionone significantly (*p* < 0.05) inhibited the decline of DPPH and ABTS radical scavenging rates of broccoli during storage (Figure 1A,B).

### 3.2. Effects of β-Ionone Treatment on Glucosinolate Content and the Expression of Its Biosynthesis Genes in Postharvest Broccoli

**Figure 2:**
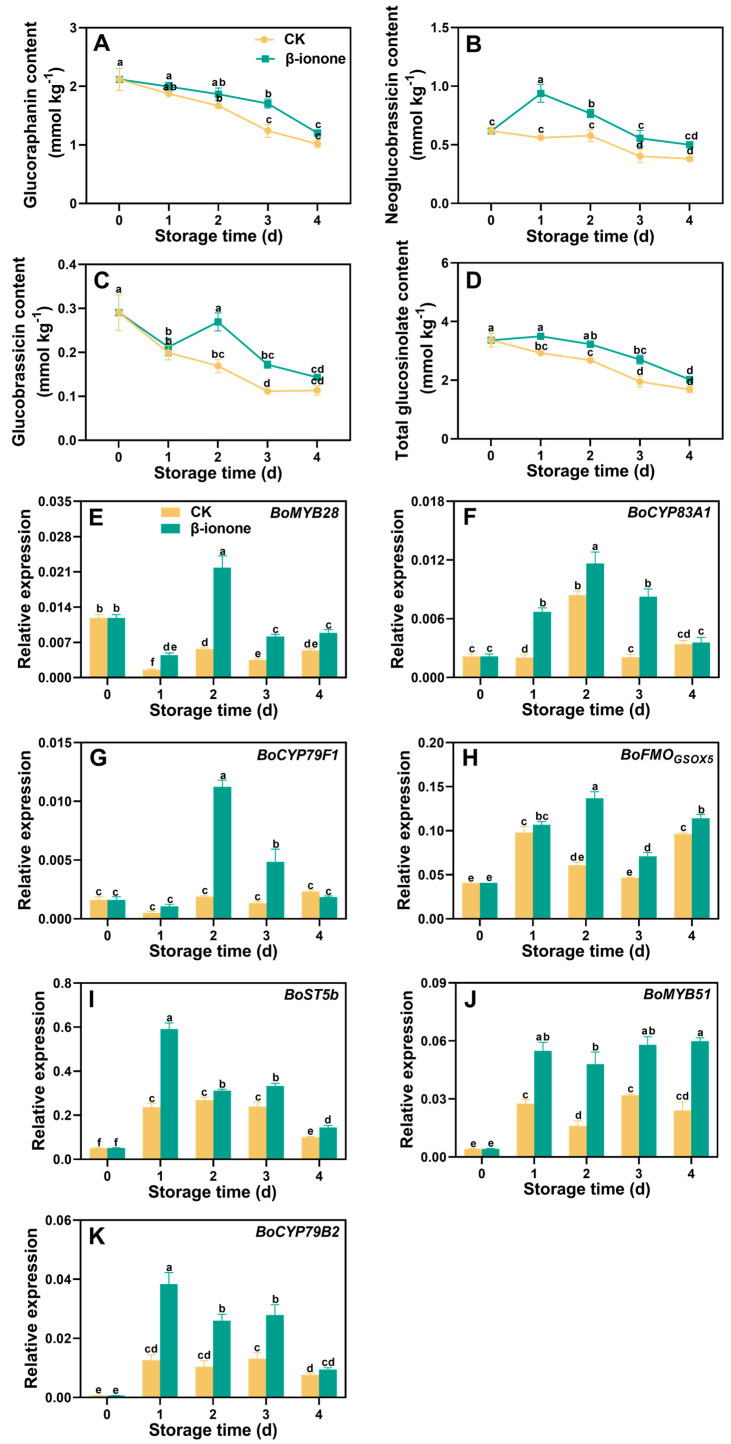
The content of three glucosinolate components (**A**–**C**) and total glucosinolate (**D**), and the expression levels of their biosynthetic genes (**E**–**K**) in broccoli treated with β-ionone and the control during storage at 15 °C. Mean values (n = 3 for (**A**–**D**), n = 4 for (**E**–**K**)), and standard deviation (vertical bars) are represented. Data points carrying different letters indicate statistically significant differences (*p* < 0.05).

The contents of GRA, NGBS, and GBS in the non-treated broccoli gradually decreased with storage. However, NGBS content in the β-ionone treated florets was consistently higher than those in the controls during storage, and the treatment also increased the other two bioactive substances, such as GRA and GBS, after two days of storage, leading to the higher total glucosinolate content in the β-ionone treated florets throughout the entire storage (Figure 2A–D). The expression levels of *BoMYB28*, *BoCYP83A1*, *BoCYP79F1*, *BoFMO_GSOX5_*, and *BoST5b* transcripts peaked on the second and first day of storage, respectively, and declined thereafter (Figure 2E–I). Treatment with β-ionone significantly (*p* < 0.05) upregulated the transcript abundances of these four genes involved in GRA synthesis during postharvest storage of broccoli. Meanwhile, the expression of transcription factor *BoMYB51* increased within the storage while key gene *BoCYP79B2* was involved in the synthesis of NGBS, and GBS increased first and then declined afterward. β-Ionone-treated broccoli displayed a pronounced upregulation of these two genes during the whole storage (Figure 2J,K).

### 3.3. Effects of β-Ionone Treatment on Riboflavin Content and the Expression of Its Biosynthesis Genes in Postharvest Broccoli

**Figure 3:**
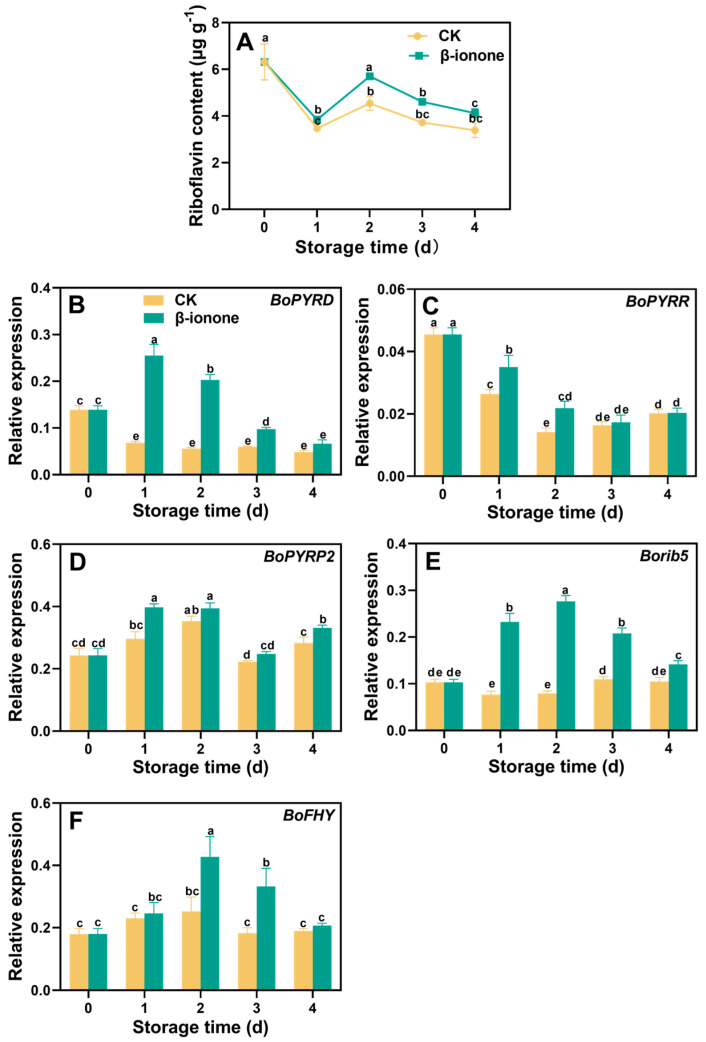
The riboflavin content (**A**) and the expression of its biosynthesis genes (**B**–**F**) in broccoli treated with β-ionone and the control during storage at 15 °C. Mean values (n = 3 for (**A**), n = 4 for (**B**–**F**)) and standard deviation (vertical bars) are represented. Data points carrying different letters indicate statistically significant differences (*p* < 0.05).

During the postharvest storage of broccoli, there was a rapid decrease in riboflavin content on the first day, followed by an increase on the second day. This increase was followed by a continued decrease in riboflavin content until the end of the storage period. However, β-ionone treatment effectively maintained its content during storage (Figure 3A). Compared to the control, the β-ionone treatment showed a significant increase (*p* < 0.05) in *BoPYRD* transcription levels throughout the storage (Figure 3B). The expression levels of *BoPYRR* showed a gradual downward trend during storage, which could be inhibited by the β-ionone treatment on the second day of storage (Figure 3C). In untreated broccoli, the expression levels of *BoPYRP2*, *Borib5*, and *BoFHY* remained relatively stable. β-ionone treatment upregulated the expression of *BoPYRP2* and *Borib5* during storage (Figure 3D,E), and higher transcripts of *BoFHY* were observed in treated florets after 2 days of storage (Figure 3F).

### 3.4. Effects of β-Ionone Treatment on AsA, GSH, and GSSG Content in Postharvest Broccoli

**Figure 4:**
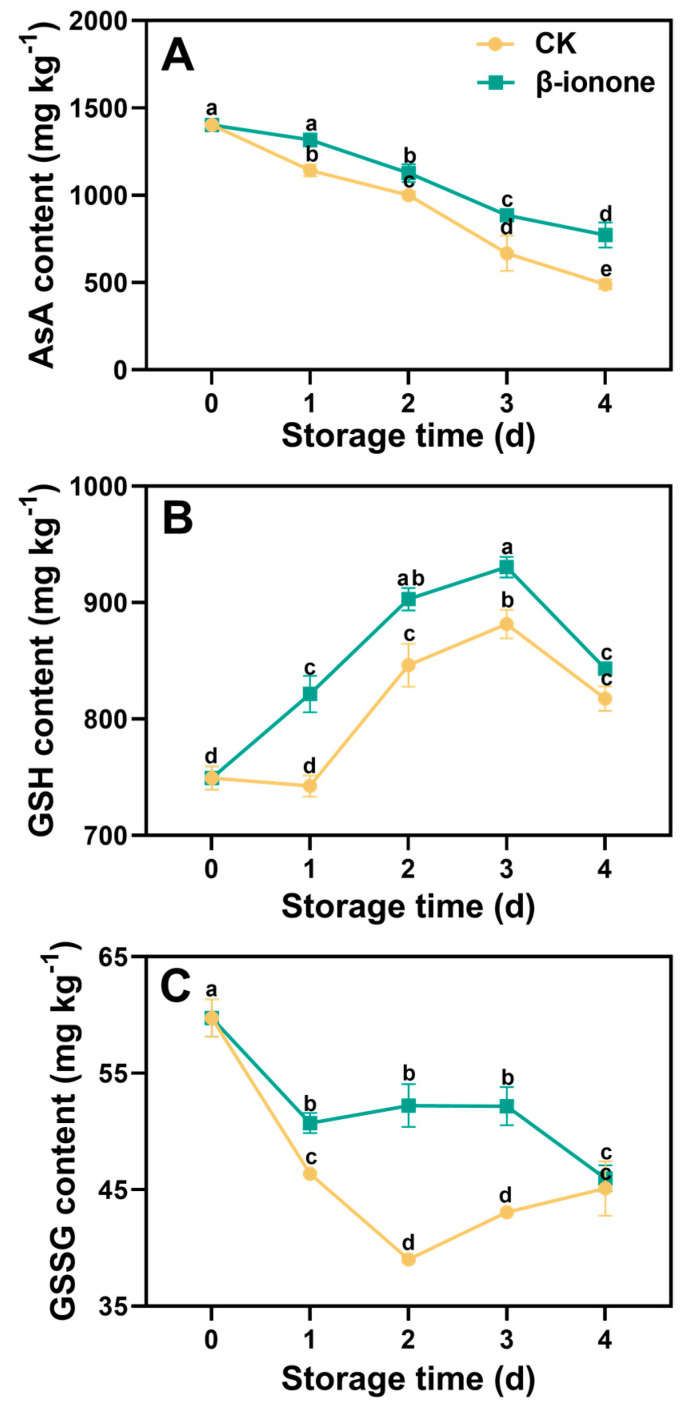
The contents of AsA (**A**), GSH (**B**), and GSSG (**C**) in broccoli treated with β-ionone and the control during storage at 15 °C. Mean values (n = 3) and standard deviation (vertical bars) are represented. Data points carrying different letters indicate statistically significant differences (*p* < 0.05).

During postharvest storage, the content of AsA in broccoli gradually decreased, which was slowed down by the β-ionone treatment (Figure 4A). During storage, the content of GSH in broccoli initially increased and then sharply decreased toward the end of storage. The content of GSH in β-ionone-treated broccoli was consistently higher than that in the non-treated controls within the storage. The content of GSSG rapidly decreased at the beginning of storage and then slightly increased. β-ionone treatment significantly (*p* < 0.05) inhibited the decline in GSSG content on the first day of storage, and the content of GSSG in β-ionone-treated broccoli remained higher than that in the controls during the first three days of storage (Figure 4B,C).

### 3.5. Effects of β-Ionone Treatment on the Expression of Genes Related to Asa and GSH in Postharvest Broccoli

**Figure 5:**
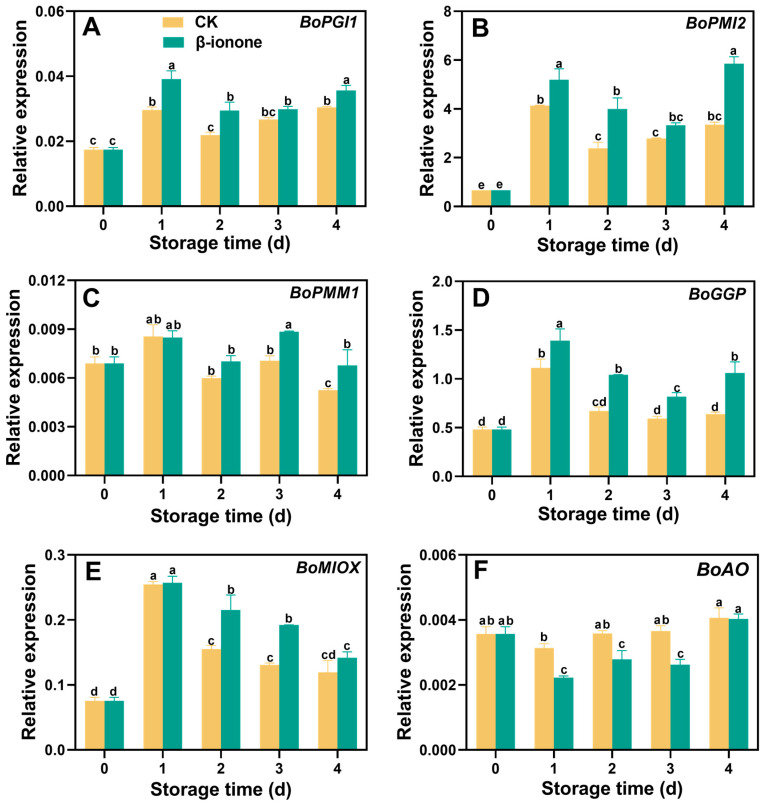
The expression levels of AsA metabolism genes (**A**–**F**) in broccoli treated with β-ionone and the control during storage at 15 °C. Mean values (n = 4) and standard deviation (vertical bars) are represented. Data points carrying different letters indicate statistically significant differences (*p* < 0.05).

**Figure 6:**
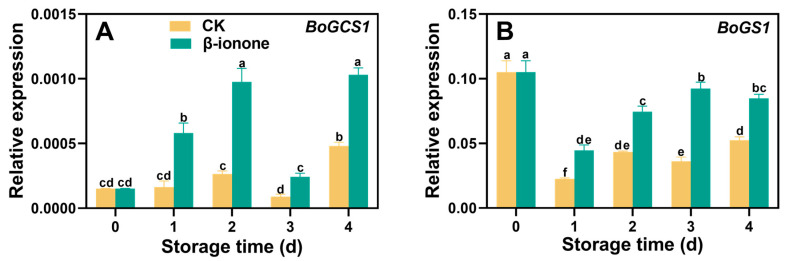
The expression levels of GSH synthesis genes (**A**–**B**) in broccoli treated with β-ionone and the control during storage at 15 °C. Mean values (n = 4) and standard deviation (vertical bars) are represented. Data points carrying different letters indicate statistically significant differences (*p* < 0.05).

**Figure 7:**
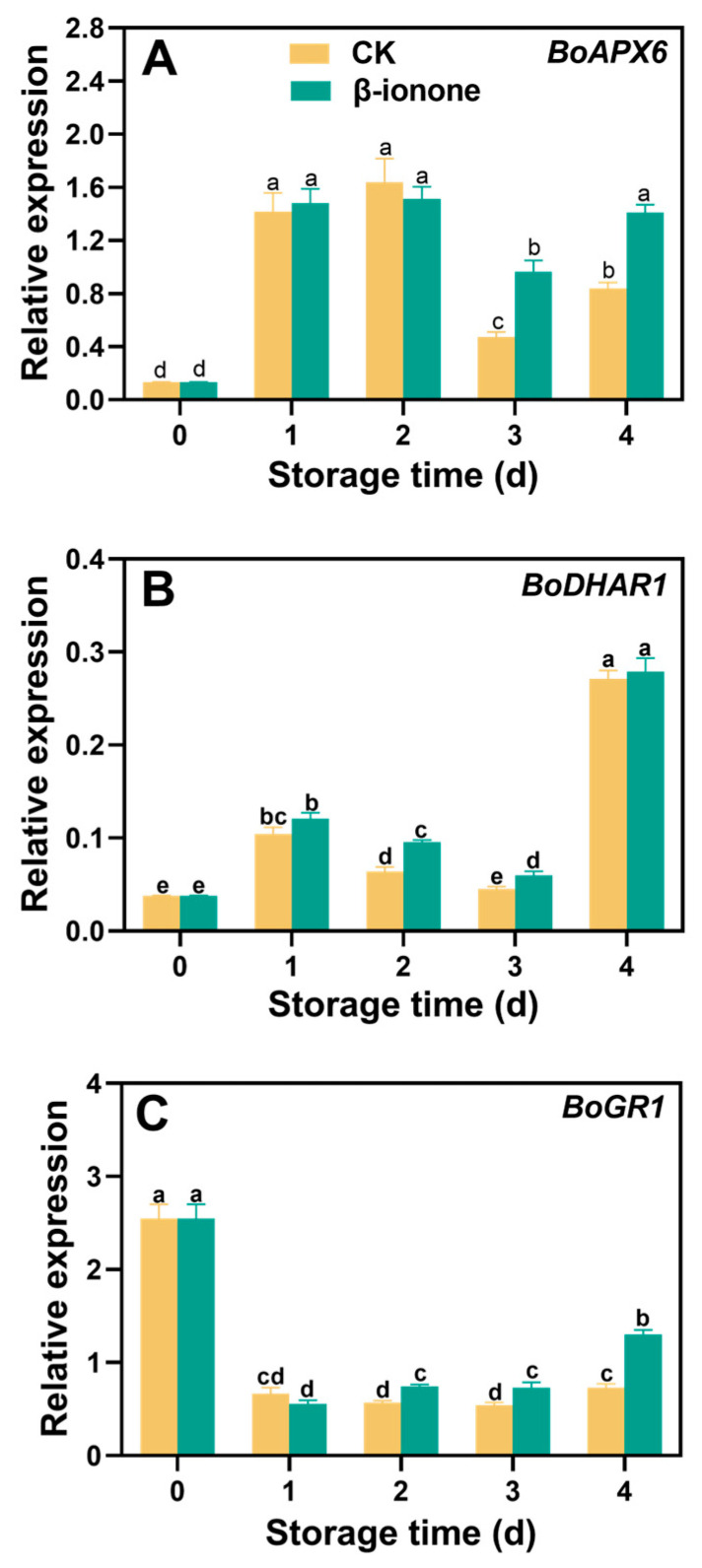
The expression levels of AsA-GSH cycle metabolism genes (**A**,**C**) in broccoli treated with β-ionone and the control during storage at 15 °C. Mean values (n = 4) and standard deviation (vertical bars) are represented. Data points carrying different letters indicate statistically significant differences (*p* < 0.05).

The expression levels of *BoPGI1* and *BoPMI2* in broccoli during postharvest storage initially increased and then decreased and finally increased at the end of storage. β-ionone treatment significantly (*p* < 0.05) upregulated the expression of *BoPGI1* and *BoPMI2* (Figure 5A,B). The expression level of *BoPMM1* remained relatively stable during storage, but β-ionone treatment increased *BoPMM1* expression after 3 days of storage (Figure 5C). The transcripts of *BoGGP* and *BoMIOX* initially increased and then decreased during storage. β-ionone treatment significantly (*p* < 0.05) enhanced *BoGGP* during storage and the upregulated *BoMIOX* on days 2 and 3 (Figure 5D,E). In untreated broccoli, the expression level of *BoAO* remained stable, but β-ionone treatment significantly (*p* < 0.05) inhibited the expression of *BoAO* during the first three days of storage (Figure 5F). Compared to the control, β-ionone treatment significantly (*p* < 0.05) upregulated the expression levels of *BoGCS1* and *BoGS1* in broccoli during postharvest storage. On the second day of storage, the transcript level of *BoGCS1* in the β-ionone-treated samples was 4.8 times higher than in the control, while *BoGS1* expression showed the greatest difference on the third day (Figure 6A,B). For *BoAPX6*, its expression in non-treated florets initially rose, then declined, and finally increased again at the end of storage. β-ionone treatment elevated *BoAPX6* transcripts after 3 days of storage (Figure 7A). The expression of *BoDHAR1* remained stable during the early to mid-storage and then surged at the end of storage. β-ionone treatment upregulated its expression during the first 3 days of storage (Figure 7B). The expression level of *BoGR1* was initially very high at the beginning of storage, decreased dramatically, and then stabilized until the end. However, β-ionone treatment significantly (*p* < 0.05) upregulated *BoGR1* expression after 2 days of storage (Figure 7C).

## 4. Discussion

It is well documented that the decline in the capacity to scavenge the ROS due to the loss of bioactive substances significantly affects its nutritional and commercial value in broccoli florets during postharvest storage [24]. Maintaining sufficient levels of bioactive substances is crucial for the antioxidant performance of broccoli after harvest. Our research revealed that treating broccoli with β-ionone significantly maintained its antioxidant capacity, as evidenced by a delayed reduction in the scavenging rates of DPPH and ABTS radicals. This novel finding indicated that exogenous β-ionone treatment could boost the antioxidant activity in postharvest broccoli, suggesting that β-ionone may influence the metabolism of bioactive compounds. β-Ionone, a regulatory metabolite derived from carotenoids, has been shown to enhance the resistance of *Arabidopsis* to *Botrytis cinerea* through the modulation of hormonal levels and the regulation of defense-related gene expression [17]. This discovery introduced β-ionone as a new member of the apocarotenoid family of hormones and regulatory metabolites, paving the way for the development of bio-fungicides that could potentially reduce the reliance on chemical fungicides. Furthermore, our results indicated that as a safe and effective method, exogenous β-ionone also played an important role in improving the nutritional quality of postharvest broccoli, which could be applied for commercial purposes.

Glucosinolates are one of the important bioactive substances in broccoli. Studies have found that the main components of glucosinolates in broccoli are GRA, NGBS, and GBS, with GRA having the highest content [4], similar to our findings. Exogenous treatments such as zinc sulfate [25], selenate [26], sucrose [27], and 1-methylcyclopropene [28] upregulated the biosynthesis of glucosinolates and successfully promoted their accumulation in broccoli. Similarly, higher levels of glucosinolates such as NGBS, GBS, and GRA were observed in the florets treated with β-ionone. Studies have shown that *MYB* transcription factors can regulate glucosinolate synthesis at the transcriptional level by modulating the expression of its target genes in broccoli. For example, *MYB28* can induce the expression of *CYP83A1* and *CYP79F1*, and *MYB51* can induce the expression of *CYP79B2*. The homologous genes of the *CYP* family can catalyze the formation of the initial substrate aldoxime for glucosinolate synthesis from related amino acids. *ST5b* is a sulfotransferase gene that functions by conjugating desulfo-glucosinolates with the sulfonate group of 3′-phosphoadenosine-5′-phosphosulfate (PAPS) to form 4-methylthiobutyl glucosinolate. *FMO_GSOX_* is responsible for the oxidation of the side chain structure to form GRA [29,30,31,32]. Additionally, studies by Wei et al. [33] and Mao et al. [34] have shown that the mixed treatment with melatonin and selenium-sulfur can upregulate the expression of *MYB28*, *CYP83A1*, *CYP79F1*, *FMO_GSOX5_*, and *ST5b*, thus promoting the production of glucosinolates and other secondary metabolites in broccoli. Wu et al. [35] demonstrated that selenium treatment can increase the transcriptional activity of *BoMYB28* and *BoMYB51* transcription factors by activating selenocysteine methyltransferase activity, thereby upregulating the expression levels of *BoCYP83A1* and *BoCYP79B2* genes, leading to increased glucosinolate production. Therefore, to explain the differences in glucosinolate content between the treated and not-treated broccoli during storage, we further studied the expression levels of genes related to its biosynthetic pathway. β-ionone treatment significantly upregulated the transcription factor *BoMYB28* and key genes *BoCYP83A1*, *BoCYP79F1*, *BoFMO_GSOX5_*, and *BoST5b*, which are closely related to the synthesis of aliphatic glucosinolates (GRA), as well as the transcription factor *BoMYB51* and key gene *BoCYP79B2* involved in the synthesis of indole glucosinolates (NGBS, GBS). Therefore, our results suggested that β-ionone treatment alleviated the loss of glucosinolates during postharvest storage by upregulating glucosinolate biosynthesis-related genes, thereby maintaining the higher antioxidant capacity in postharvest broccoli.

Riboflavin plays a crucial role in plants as a precursor to the coenzyme factors FMN and FAD, participating in mitochondrial electron transport, photosynthesis, protein folding, and other physiological and biochemical processes [36], thereby alleviating biotic and abiotic stresses in plants [37]. Studies have shown that in plants, pyrimidine deaminase, reductase, and phosphatase, decoded by the genes *PYRD*, *PYRR*, and *PYRP2*, respectively, catalyze the conversion of precursors guanosine triphosphate (GTP) and ribulose-5-phosphate (Ru5P) into 6,7-dimethyl-8-ribityllumazine, which is involved in the riboflavin synthesis [38,39,40]. The *rib5* gene regulates the production of riboflavin synthase; yeast lacking this gene showed no detectable riboflavin synthase activity, while overexpression of the *rib5* gene in yeast resulted in riboflavin synthase activity up to 20 times higher than that of wild-type yeast [41]. In plants, riboflavin can be converted to FMN and then to FAD [42]. FMN hydrolase, encoded by the *FHY* gene, catalyzes the conversion of FMN back to riboflavin, forming part of the riboflavin cycle in plants [43]. The results demonstrated that β-ionone treatment led to the upregulation of riboflavin-synthesis-related genes, including *BoPYRD*, *BoPYRR*, *BoPYRP2*, and Borib5, as well as the FMN hydrolase gene *BoFHY*, contributing to the higher riboflavin content in the β-ionone-treated florets compared to the controls, which played a role in maintaining the antioxidant performance.

AsA is a crucial antioxidant in plants, and its reduction indicates plant senescence [44]. AsA synthesis primarily occurs through four pathways: the L-galactose pathway, the D-galacturonic acid pathway, the glucose pathway, and the myoinositol pathway, with the L-galactose pathway being the most significant [45]. In this study, four key genes involved in the L-galactose pathway (*BoPGI1*, *BoPMI2*, *BoPMM1*, and *BoGGP*) were notably upregulated in broccoli treated with β-ionone. Zhang et al. [46] found that H_2_O_2_ treatment of broccoli upregulated three structural genes of the L-galactose pathway, such as *BoVTC2_5*, *BoIPP*, and *BoGalDH*, while enhancing genes related to the glucose pathway only in the later stages of storage, thus maintaining AsA content. Ascorbate oxidase (AO) is negatively correlated with the AsA content as demonstrated in kiwifruit treated with γ-aminobutyric acid [47] and in mangoes [48]. Similarly, in our study, β-ionone treatment alleviated the degradation of AsA in broccoli by inhibiting the expression of *BoAO*.

Glutathione exists in plants in two forms: GSH and GSSG. It plays a crucial role in stress resistance and antioxidation during abiotic stress and ROS production [49]. In plants, the synthesis of reduced glutathione (GSH) is primarily catalyzed by two enzymes: γ-glutamylcysteine synthetase (GCS) and glutathione synthetase (GS) [50]. Our results show that β-ionone treatment significantly upregulated the expression of *BoGCS1* and *BoGS1*, leading to higher contents of GSH and GSSG compared to the control group. Similar results were observed by Xing et al. [51], where SO_2_ fumigation of grapes increased their resistance to gray mold by upregulating the expression of *Vvγ-ECS* and *VvGS* genes, thereby enhancing the activity of γ-glutamylcysteine synthetase and glutathione synthetase and increasing the GSH content.

The combined action of AsA and GSH results in the formation of the ASA-GSH cycle, which primarily alleviates oxidative stress in plants by removing H_2_O_2_ [52]. In this cycle, the enzymes ascorbate peroxidase (APX), dehydroascorbate reductase (DHAR), and glutathione reductase (GR) play key roles. APX decomposes H_2_O_2_ in plant chloroplasts using AsA as a substrate. Dehydroascorbate produced in this process is reduced to AsA by DHAR using GSH as a substrate. GR maintains GSH in its reduced state by converting GSSG to GSH, enhancing the plant’s redox capacity [53]. Our results indicated that β-ionone treatment effectively upregulated the expression of *BoAPX6*, *BoDHAR1*, and *BoGR1*, potentially enhancing the ASA-GSH cycle in broccoli. Previous studies have confirmed that the accumulation of DHAR and GR increased the content of AsA and GSH in plants in rice [54] and barley [55]. Taken together, our results suggested that β-ionone treatment increased the levels of AsA and GSH in postharvest broccoli by regulating related genes involved in their biosynthesis and cycling. In conclusion, the β-ionone treatment delayed the decline in antioxidant activity of postharvest broccoli, which was evidenced by the increase in levels of AsA and GSH.

## 5. Conclusions

In conclusion, our study showed that β-ionone treatment maintained the antioxidant capacity due to the higher levels of bioactive substances such as glucosinolates, riboflavin, AsA, and GSH in postharvest broccoli florets. The upregulation of the expression of several relevant biosynthetic genes in the treated florets was associated with the increased contents of glucosinolate and riboflavin. Meanwhile, the treatment also positively regulated AsA and GSH-related genes, thereby maintaining the levels of these two non-enzymatic antioxidant substances. Therefore, our findings suggest that β-ionone treatment represents a novel and viable technology for the postharvest preservation of broccoli, offering significant potential for improving its quality after harvest. It is our understanding that this is the first report demonstrating that exogenous β-ionone treatment can enhance nutritional values in postharvest horticultural products.

## Data Availability

The original contributions presented in the study are included in the article/Supplementary Materials; further inquiries can be directed to the corresponding authors.

## Supplementary Materials

The following supporting information can be downloaded at: https://www.mdpi.com/article/10.3390/foods14050762/s1, Table S1: Primer sequences used for RT-qPCR analysis. Table S2: Data normality test. Table S3: Data Homogeneity of Variances Test. Table S4: Statistical significance test of data differences. Figure S1: The photographic records of broccoli with different treatments during postharvest storage.

## Abbreviations

The following abbreviations are used in this manuscript:

DPPH	1,1-Diphenyl-2-picrylhydrazyl
ABTS	2,2′-Azinobis-(3-ethylbenzthiazoline-6-sulphonate)
AsA	Ascorbic acid
GSH	Glutathione
GRA	Glucoraphanin
GBS	Glucobrassicin
NGBS	Neoglucobrassicin
FMN	Flavin mononucleotide
FAD	Flavin adenine dinucleotide

## References

[1] Li H., Xia Y., Liu H.-Y., Guo H., He X.-Q., Liu Y., Wu D.-T., Mai Y.-H., Li H.-B., Zou L. (2022). Nutritional Values, Beneficial Effects, and Food Applications of Broccoli (*Brassica oleracea* Var. *Italica Plenck*). Trends Food Sci. Technol..

[2] Xu F., Wang H., Tang Y., Dong S., Qiao X., Chen X., Zheng Y. (2016). Effect of 1-Methylcyclopropene on Senescence and Sugar Metabolism in Harvested Broccoli Florets. Postharvest Biol. Technol..

[3] Zhao J., Zhang X., Li F., Lei X., Ge L., Li H., Zhao N., Ming J. (2024). The Effects of Interventions with Glucosinolates and Their Metabolites in Cruciferous Vegetables on Inflammatory Bowel Disease: A Review. Foods.

[4] Yang Q., Zhou Q., Zhou X., Fang H., Zhao Y., Wei B., Ji S. (2022). Insights into Profiling of Glucosinolates and Genes Involved in Its Metabolic Pathway Accompanying Post-Harvest Yellowing of Broccoli. Postharvest Biol. Technol..

[5] Jiadkong K., Fauzia A.N., Yamaguchi N., Ueda A. (2024). Exogenous Riboflavin (Vitamin B2) Application Enhances Salinity Tolerance through the Activation of Its Biosynthesis in Rice Seedlings under Salinity Stress. Plant Sci..

[6] Proietti S., Moscatello S., Famiani F., Battistelli A. (2009). Increase of Ascorbic Acid Content and Nutritional Quality in Spinach Leaves during Physiological Acclimation to Low Temperature. Plant Physiol. Biochem..

[7] Piechowiak T. (2021). Effect of Ozone Treatment on Glutathione (GSH) Status in Selected Berry Fruit. Phytochemistry.

[8] Raseetha S., Leong S.Y., Burritt D.J., Oey I. (2013). Understanding the Degradation of Ascorbic Acid and Glutathione in Relation to the Levels of Oxidative Stress Biomarkers in Broccoli (*Brassica oleracea* L. *Italica* Cv. *Bellstar*) during Storage and Mechanical Processing. Food Chem..

[9] Cabello-Hurtado F., Gicquel M., Esnault M.-A. (2012). Evaluation of the Antioxidant Potential of Cauliflower (*Brassica oleracea*) from a Glucosinolate Content Perspective. Food Chem..

[10] Ciudad-Mulero M., Domínguez L., Morales P., Fernández-Ruiz V., Cámara M. (2023). A Review of Foods of Plant Origin as Sources of Vitamins with Proven Activity in Oxidative Stress Prevention According to EFSA Scientific Evidence. Molecules.

[11] Wang L., Wang F., Zhang Y., Ma Y., Guo Y., Zhang X. (2020). Enhancing the Ascorbate–Glutathione Cycle Reduced Fermentation by Increasing NAD+ Levels during Broccoli Head Storage under Controlled Atmosphere. Postharvest Biol. Technol..

[12] Wei H., Li Y., Wei L., Peng S., Zhang B., Xu D., Cheng X. (2024). Exploring the Mechanism of Exopolysaccharides in Mitigating Cadmium Toxicity in Rice through Analyzing the Changes of Antioxidant System. J. Hazard. Mater..

[13] Ansari M., Emami S. (2016). β-Ionone and Its Analogs as Promising Anticancer Agents. Eur. J. Med. Chem..

[14] Lalko J., Lapczynski A., McGinty D., Bhatia S., Letizia C.S., Api A.M. (2007). Fragrance Material Review on β-Ionone. Food Chem. Toxicol..

[15] Xie H., Liu T., Chen J., Yang Z., Xu S., Fan Y., Zeng J., Chen Y., Ma Z., Gao Y. (2019). Activation of PSGR with β-Ionone Suppresses Prostate Cancer Progression by Blocking Androgen Receptor Nuclear Translocation. Cancer Lett..

[16] Miranda M.L.P., Furtado K.S., De Oliveira Andrade F., Heidor R., Da Cruz R.S., Nogueira M.S., De Castro I.A., Purgatto E., Barbisan L.F., Moreno F.S. (2019). β-Ionone Inhibits Nonalcoholic Fatty Liver Disease and Its Association with Hepatocarcinogenesis in Male Wistar Rats. Chem. Biol. Interact..

[17] Felemban A., Moreno J.C., Mi J., Ali S., Sham A., AbuQamar S.F., Al-Babili S. (2024). The Apocarotenoid Β-ionone Regulates the Transcriptome of *Arabidopsis thaliana* and Increases Its Resistance against *Botrytis cinerea*. Plant J..

[18] Suo K., Zhang Y., Feng Y., Yang Z., Zhou C., Chen W., Wang J. (2023). Ultrasonic Synergistic Slightly Acidic Electrolyzed Water Processing to Improve Postharvest Storage Quality of Chinese Bayberry. Ultrason. Sonochem..

[19] Brown P.D., Tokuhisa J.G., Reichelt M., Gershenzon J. (2003). Variation of Glucosinolate Accumulation among Different Organs and Developmental Stages of Arabidopsis Thaliana. Phytochemistry.

[20] Chen C.-H., Yang Y.-H., Shen C.-T., Lai S.-M., Chang C.-M.J., Shieh C.-J. (2011). Recovery of Vitamins B from Supercritical Carbon Dioxide-Defatted Rice Bran Powder Using Ultrasound Water Extraction. J. Taiwan Inst. Chem. Eng..

[21] Zohora F.-T., Sarwar S., Khatun O., Begum P., Khatun M., Ahsan M., Nazrul Islam S. (2020). Estimation of B-Vitamins (B1, B2, B3 and B6) by HPLC in Vegetables Including Ethnic Selected Varieties of Bangladesh. Pharm. Pharmacol. Int. J..

[22] Zhou C., Dong W., Jin S., Liu Q., Shi L., Cao S., Li S., Chen W., Yang Z. (2022). γ-Aminobutyric Acid Treatment Induced Chilling Tolerance in Postharvest Peach Fruit by Upregulating Ascorbic Acid and Glutathione Contents at the Molecular Level. Front. Plant Sci..

[23] Yan W., Cao M., Shi L., Wu W., Xu F., Chen W., Yang Z. (2024). γ-Aminobutyric Acid Delays Fruit Softening in Postharvest Kiwifruit by Inhibiting Starch and Cell Wall Degradation. Postharvest Biol. Technol..

[24] Dos Reis L.C.R., De Oliveira V.R., Hagen M.E.K., Jablonski A., Flôres S.H., De Oliveira Rios A. (2015). Carotenoids, Flavonoids, Chlorophylls, Phenolic Compounds and Antioxidant Activity in Fresh and Cooked Broccoli (*Brassica oleracea* Var. *Avenger*) and Cauliflower (*Brassica oleracea* Var. *Alphina F1*). LWT Food Sci. Technol..

[25] Rao S., Gou Y., Yu T., Cong X., Gui J., Zhu Z., Zhang W., Liao Y., Ye J., Cheng S. (2021). Effects of Selenate on Se, Flavonoid, and Glucosinolate in Broccoli Florets by Combined Transcriptome and Metabolome Analyses. Food Res. Int..

[26] Yang R., Guo L., Jin X., Shen C., Zhou Y., Gu Z. (2015). Enhancement of Glucosinolate and Sulforaphane Formation of Broccoli Sprouts by Zinc Sulphate via Its Stress Effect. J. Funct. Foods.

[27] Xu F., Tang Y., Dong S., Shao X., Wang H., Zheng Y., Yang Z. (2016). Reducing Yellowing and Enhancing Antioxidant Capacity of Broccoli in Storage by Sucrose Treatment. Postharvest Biol. Technol..

[28] Yuan G., Sun B., Yuan J., Wang Q. (2010). Effect of 1-Methylcyclopropene on Shelf Life, Visual Quality, Antioxidant Enzymes and Health-Promoting Compounds in Broccoli Florets. Food Chem..

[29] Li C., Song S., He Y., Liu H. (2024). Transcriptomics Integrated with Metabolomics Reveals the Mechanism of CaCl_2_-HCl Electrolyzed Water-Induced Glucosinolate Biosynthesis in Broccoli Sprouts. Food Sci. Hum. Wellness.

[30] Han Y., He X., Luo S., Hu H., Li P. (2024). The Regulatory Effect of Slightly Acidic Electrolyzed Water Ice on the Postharvest Quality Decline and Glucosinolate Metabolism of Broccoli. Postharvest Biol. Technol..

[31] Casajús V., Howe K., Fish T., Civello P., Thannhauser T., Li L., Gómez Lobato M., Martínez G. (2023). Evidence of Glucosinolates Translocation from Inflorescences to Stems during Postharvest Storage of Broccoli. Plant Physiol. Biochem..

[32] Hassini I., Rios J.J., Garcia-Ibañez P., Baenas N., Carvajal M., Moreno D.A. (2019). Comparative Effect of Elicitors on the Physiology and Secondary Metabolites in Broccoli Plants. J. Plant Physiol..

[33] Wei L., Liu C., Zheng H., Zheng L. (2020). Melatonin Treatment Affects the Glucoraphanin-Sulforaphane System in Postharvest Fresh-Cut Broccoli (*Brassica oleracea* L.). Food Chem..

[34] Mao S., Wang J., Wu Q., Liang M., Yuan Y., Wu T., Liu M., Wu Q., Huang K. (2020). Effect of Selenium–Sulfur Interaction on the Anabolism of Sulforaphane in Broccoli. Phytochemistry.

[35] Wu Q., Wang J., Tian Y., Zhou C., Mao S., Wu Q., Huang K. (2023). Selenocysteine Methyltransferase SMT Catalyzed the Synthesis of Se-Methylselenocysteine to Regulate the Accumulation of Glucosinolates and Sulforaphane in Broccoli. Hortic. Plant J..

[36] Higa A., Khandakar J., Mori Y., Kitamura Y. (2012). Increased de Novo Riboflavin Synthesis and Hydrolysis of FMN Are Involved in Riboflavin Secretion from Hyoscyamus Albus Hairy Roots under Iron Deficiency. Plant Physiol. Biochem..

[37] Cao D., Heughebaert L., Boffel L., Stove C., Van Der Straeten D. (2024). Simultaneous Quantification of Seven B Vitamins from Wheat Grains Using UHPLC-MS/MS. Food Chem..

[38] Sa N., Rawat R., Thornburg C., Walker K.D., Roje S. (2016). Identification and Characterization of the Missing Phosphatase on the Riboflavin Biosynthesis Pathway. Arabidopsis Thaliana. Plant J..

[39] Wang T., Wang J., Zhang D., Chen L., Liu M., Zhang X., Schmidt W., Zhang W. (2023). Protein Kinase MtCIPK12 Modulates Iron Reduction in *Medicago truncatula* by Regulating Riboflavin Biosynthesis. Plant Cell Environ..

[40] Zhou G., Jiang W., Luo H., Li X., Wan Y. (2023). Transcriptome and Targeted Metabolomic Integrated Analysis Reveals Mechanisms of B Vitamin Accumulation in Areca Catechu Nut Development. Int. J. Biol. Macromol..

[41] Santos M.A., García-Ramírez J.J., Revuelta J.L. (1995). Riboflavin Biosynthesis in Saccharomyces Cerevisiae. J. Biol. Chem..

[42] Lynch J.H., Roje S. (2022). A Higher Plant FAD Synthetase Is Fused to an Inactivated FAD Pyrophosphatase. J. Biol. Chem..

[43] Rawat R., Sandoval F.J., Wei Z., Winkler R., Roje S. (2011). An FMN Hydrolase of the Haloacid Dehalogenase Superfamily Is Active in Plant Chloroplasts. J. Biol. Chem..

[44] Zhang Y., Wang K., Xiao X., Cao S., Chen W., Yang Z., Shi L. (2021). Effect of 1-MCP on the Regulation Processes Involved in Ascorbate Metabolism in Kiwifruit. Postharvest Biol. Technol..

[45] Dowdle J., Ishikawa T., Gatzek S., Rolinski S., Smirnoff N. (2007). Two Genes in *Arabidopsis thaliana* Encoding GDP-L-galactose Phosphorylase Are Required for Ascorbate Biosynthesis and Seedling Viability. Plant J..

[46] Zhang Y., Chen Y., Guo Y., Sun Y., Wang Z., Wang Y., Guan L., Wang L., Zhou Q. (2023). Transcriptome and Metabolome Integrated Analysis Revealed the Effects and Potential Mechanism of Hydrogen Peroxide on Antioxidant System in Postharvest Broccoli. Postharvest Biol. Technol..

[47] Liu Q., Li X., Jin S., Dong W., Zhang Y., Chen W., Shi L., Cao S., Yang Z. (2023). γ-Aminobutyric Acid Treatment Induced Chilling Tolerance in Postharvest Kiwifruit (*Actinidia chinensis* Cv. *Hongyang*) via Regulating Ascorbic Acid Metabolism. Food Chem..

[48] Wang L., Li R., Shi X., Wei L., Li W., Shao Y. (2023). Ripening Patterns (off-Tree and on-Tree) Affect Physiology, Quality, and Ascorbic Acid Metabolism of Mango Fruit (Cv. *Guifei*). Sci. Hortic..

[49] Yu G.-B., Tian J., Chen R.-N., Liu H.-L., Wen B.-W., Wei J.-P., Chen Q., Chen F., Sheng Y., Yang F.-J. (2023). Glutathione-Dependent Redox Homeostasis Is Critical for Chlorothalonil Detoxification in Tomato Leaves. Ecotoxicol. Environ. Saf..

[50] Noctor G., Mhamdi A., Chaouch S., Han Y., Neukermans J., Marquez-Garcia B., Queval G., Foyer C.H. (2012). Glutathione in Plants: An Integrated Overview. Plant Cell Environ..

[51] Xing S., Wang M., Zhang Z., Yuan Y., Song Z., Wu B., Wei J. (2024). Sulfur Dioxide Enhances Postharvest Grape Resistance to Botrytis Cinerea by Promoting Glutathione Level. Sci. Hortic..

[52] Lu X., Zhang H., Zhang N., Dong C., Ji H., Yu J., Ban Z., Yan R., Zhang T., Chen C. (2023). Effects of Ozone Treatment on Gene Profiling Involved in ASA-GSH Cycle in Postharvest Cantaloupe. Sci. Hortic..

[53] Zhou F., Xu D., Xiong S., Chen C., Liu C., Jiang A. (2024). Inhibition of Wound Healing in Fresh-Cut Potatoes by Ascorbic Acid Is Associated with Control of the Levels of Reactive Oxygen Species and the AsA-GSH Cycle. Sci. Hortic..

[54] Altaf M.M., Diao X., Altaf M.A., Ur Rehman A., Shakoor A., Khan L.U., Jan B.L., Ahmad P. (2022). Silicon-Mediated Metabolic Upregulation of Ascorbate Glutathione (AsA-GSH) and Glyoxalase Reduces the Toxic Effects of Vanadium in Rice. J. Hazard. Mater..

[55] Cheng C., Zhao X., Yang H., Coldea T.E., Zhao H. (2023). Mechanism of Selenite Tolerance during Barley Germination: A Combination of Tissue Selenium Metabolism Alterations and Ascorbate-Glutathione Cycle Modulation. Plant Physiol. Biochem..

