# Supplementary material for: β-Ionone Treatment Enhances the Antioxidant Capacity in Postharvest Broccoli (Brassica oleracea L. var. Italica) by Maintaining the Levels of Bioactive Substances
Source: Foods. 2025 Feb 24;14(5):762. doi: 10.3390/foods14050762 (PMC11898509; doi:10.3390/foods14050762)
Supplement: Supplementary file 1 [file foods-14-00762-s001.zip › supplementary table s1.pdf]

Table S1. Primer sequences used for RT-qPCR analysis

Gene name	Forward Primer	Reverse Primer
BoActin	CGGAATGGTCAAGGCTGGTTTC	CCATGTCATCCCAGTTGCTCAC
BoMYB28	CTGAGAATGAGATGAATACT	GACACATAGGACATAAGAA
BoCYP83A1	TCAAGACGCAAGACGTCAAC	CAAGTGGTTCATCCCCATCT
BoCYP79F1	TCCGATGGTTCTCATGTTGA	AACCGGATATCGCATGTTTC
BoFMO$_{GSOX5}$	TAATGGACACTACACAGA	TTCCTATCACTACCACAA
BoST5b	CCCATATACCCAACGGGTCG	CCCATGAACTCAGCCAACCT
BoMYB51	CCCAGAGATTCCAGAGAAGC	CCGATAATCTCAGACAGAACGC
BoCYP79B2	GCACAGAACGTCCTCTCTAAC	AATGAAGCCACCTGTGTCTCGCA
BoPYRD	TCTTCTTGATGTTGAGTT	GGCTCTTATCTTCTTGTA
BoPYRR	GTGGAGCATTACTATCAG	CATTACTTCTATCTTCACATC
BoPYRP2	TTGTGAATGTCTTGATGA	AACTTCTTTATCCCTATGG
Borib5	CTTCACTCAGACCAAATC	CAATCCCAGATCCTTTAC
BoFHY	TCCTGAGAATAATGAGAAG	AGAAGATAACACGCAATA
BoPGI1	GTCCATTAGTGTTACCAT	CATTAAGAACTGACAATACA
BoPMI2	CTATGCTCTATGCTTACC	CCTGTTCCTTCTATAACC
BoPMM1	GAAATGGAATGCTCAATG	GAAAGTAAGGTTAAGATGTG
BoGGP	GAGATGAAGAAGAAGGTT	TATGATGTCTATGCTGTC
BoMIOX	TGGTCTCTTCATTATTCG	GTCTTCATCGTTCATCAA
BoAO	GTATTCCGACGATTATTCT	GCTGCTTATGTTCTGTAG
BoGCS1	CATCATCATCATCATCATC	AACCTACCAGTATCTTGA
BoGS1	ACTGGATGTTCCTATGTA	ATCTCTTCAACCTAACCT
BoAPX6	GTAATGTTGTCCACTGTA	ACTCTCCTTATCTCATCTT
BoDHAR1	GGTTCTTAGCCATTAGTC	GAGTCTTTGCTCTTCAAG
BoGR1	CTACTTATGGTGGTGAAC	CTCAATATCTCATCAGTCTT
