# Supplementary figures and images for: β-Ionone Treatment Enhances the Antioxidant Capacity in Postharvest Broccoli (Brassica oleracea L. var. Italica) by Maintaining the Levels of Bioactive Substances
Source: Foods. 2025 Feb 24;14(5):762. doi: 10.3390/foods14050762 (PMC11898509; doi:10.3390/foods14050762)

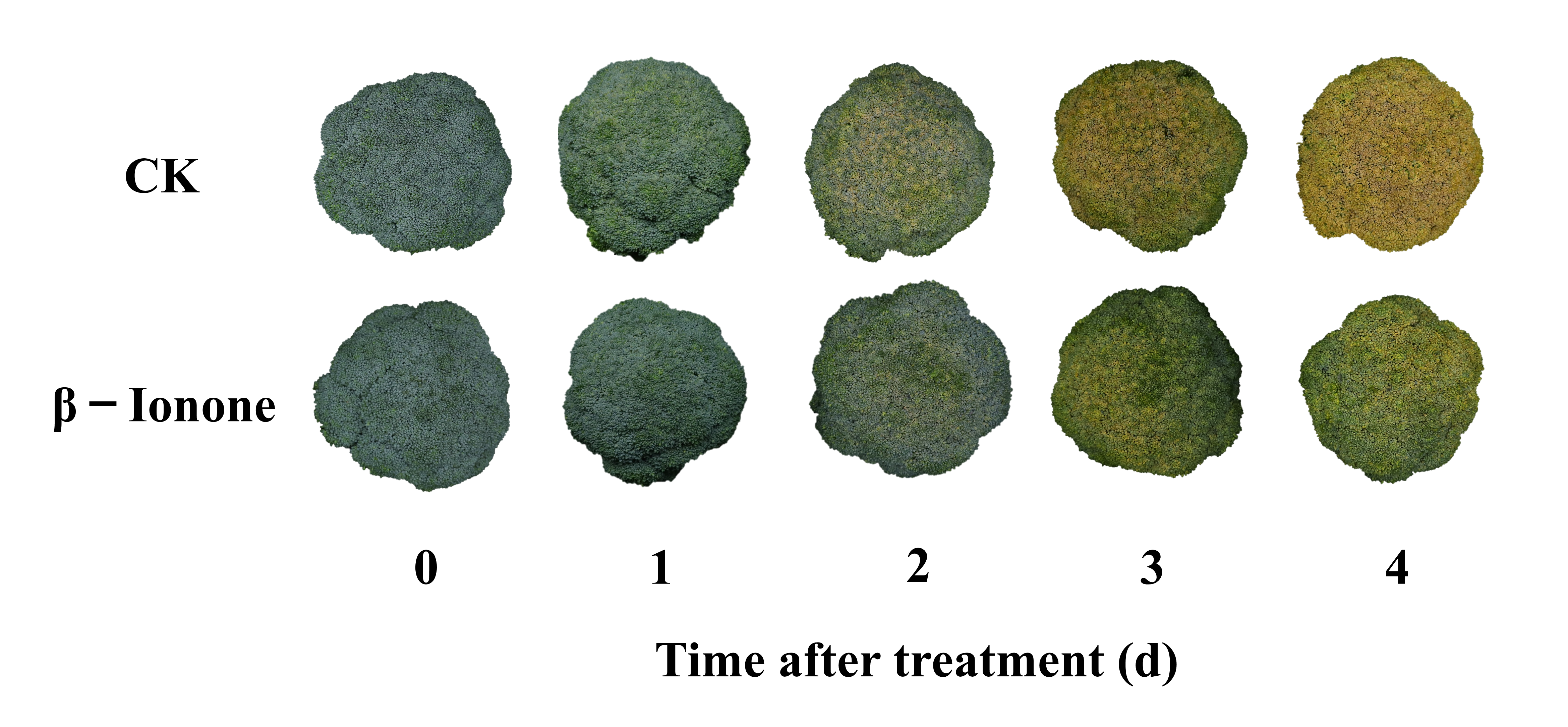

Supplement: Supplementary file 1 [file foods-14-00762-s001.zip › supplementary figure s1.tif]
